# Insights into the Intraspecific Variability of the above and Belowground Emissions of Volatile Organic Compounds in Tomato

**DOI:** 10.3390/molecules26010237

**Published:** 2021-01-05

**Authors:** Nafissa Dehimeche, Bruno Buatois, Nadia Bertin, Michael Staudt

**Affiliations:** 1 Centre d’Ecologie Fonctionnelle et Evolutive, CNRS-Université Montpellier-Université Paul-Valéry Montpellier–EPHE, Campus CNRS, CEDEX 5, F-34293 Montpellier, France; nafissa.dehimeche@cefe.cnrs.fr (N.D.); bruno.buatois@cefe.cnrs.fr (B.B.); 2 INRAE, UR115 Plantes et Systèmes de Culture Horticoles, Site Agroparc, 84914 Avignon, France; nadia.bertin@inrae.fr

**Keywords:** aboveground-belowground interactions, biomarkers, chemodiversity, isoprene, monoterpene, salicylate, sesquiterpene, *Solanum lycopersicum*, volatile organic compound emission

## Abstract

The in-vivo monitoring of volatile organic compound (VOC) emissions is a potential non-invasive tool in plant protection, especially in greenhouse cultivation. We studied VOC production from above and belowground organs of the eight parents of the Multi-Parent Advanced Generation Intercross population (MAGIC) tomato population, which exhibits a high genetic variability, in order to obtain more insight into the variability of constitutive VOC emissions from tomato plants under stress-free conditions. Foliage emissions were composed of terpenes, the majority of which were also stored in the leaves. Foliage emissions were very low, partly light-dependent, and differed significantly among genotypes, both in quantity and quality. Soil with roots emitted VOCs at similar, though more variable, rates than foliage. Soil emissions were characterized by terpenes, oxygenated alkanes, and alkenes and phenolic compounds, only a few of which were found in root extracts at low concentrations. Correlation analyses revealed that several VOCs emitted from foliage or soil are jointly regulated and that above and belowground sources are partially interconnected. With respect to VOC monitoring in tomato crops, our results underline that genetic variability, light-dependent de-novo synthesis, and belowground sources are factors to be considered for successful use in crop monitoring.

## 1. Introduction

Like all living organisms, plants exchange a huge number of volatile metabolites with their environment at their aboveground and belowground organs [1]. More than 1700 organic gases, collectively referred to as Volatile Organic Compounds (VOCs), are currently known to be produced and released by plants. The majority are terpenoïds, phenolic compounds (benzenoïds, phenyl-propanoïdes), and derivatives of fatty and amino acids. Especially under detrimental life conditions and biotic aggressions, plants release a large variety of stress-induced VOCs, whose blend depends on the type and intensity of stress [2] and the inherent capacity of the plant to produce these [3]. Stress-induced VOCs are not only simple byproducts of tissue damages, but many of them are produced by specialized enzymes that are activated in response to the aggressors as part of a local or systemic reaction cascade [4]. Numerous studies have demonstrated the roles of VOCs in plant defense, acting either directly by repelling biotic stressors or indirectly by attracting natural enemies [5]. In addition, VOCs that are released into the atmosphere or soil are involved in within-plant and between plant stress signaling [6,7]. Plant VOCs also affect the food web by serving as cues for herbivores to localize their host plants, or as a food source for microbes in both the rhizosphere and phyllosphere [8,9,10].

Owing to their multiple roles in plant ecology and advances in the real-time detection of trace gases (e.g., [11,12]), the study of VOCs that are emitted by crop plants has received increasing attention in the field of agroecology and crop protection. Provided that the emitted VOCs can be unequivocally identified and quantitatively measured without disturbing the actual physiological status of the plants, the online monitoring of the volatile metabolome represents a valuable non-invasive tool for early stress detection and survey of variation in plant fitness and phenology [13,14,15]. Greenhouse crops are particularly suitable for this application, due to the lower and more constant air exchange than in the field, which favors the accumulation of trace gases in the air to values that are distinguishable from the background [16]. In recent years, several pilot studies on tomato have proven the potential usefulness of VOC monitoring, but also revealed limitations and obstacles to overcome [11,17,18,19,20]. One prerequisite for a successful application is a sound knowledge of the natural variability of VOC release under non-stressed conditions. Yet, establishing an accurate reference baseline of VOC emission is not straightforward, mainly due to the possible heterogeneity of VOC sources and pitfalls in the measurement methods: first, the quantity and composition of VOCs may vary among cultivars [21,22]. Second, root emissions and VOC exchanges at the soil/air interface can blur the chemical messages that are released by the foliage and vice-versa. Third, tomato leaves and stem are more or less densely covered by glandular trichomes [23,24], whose tips contain large amounts of VOCs. Even tiny movements and mechanical disturbance can cause large emission bursts without any relation to biotic stress [18,25]. Fourth, the actual emission rate also depends on environmental factors, such as temperature, light conditions, atmospheric CO_2_, and soil properties, whose potential influences must be considered during measurements and data interpretation [26,27,28].

In order to gain more insight and provide new references for further studies, we investigated the genetic variability of constitutive VOC production in vegetative tomato plants (*Solanum lycopersicum* L.). We performed a screening study on the eight parents (accessions) of the MAGIC tomato populations (Multi-Parent Advanced Generation Intercross population of Tomato) representing a large genetic variability [29]. This population presents an exceptional wide range of genetic variation, a saturated genetic map, and it is particularly amenable to QTL detection and causal polymorphism identification [29,30]. These parents include four genotypes from the *Solanum lycopersicum lycopersicum* group with large fruits and four genotypes from the *Solanum lycopersicum cerasiforme* group with cherry-type fruits. VOC emissions were measured quantitatively under environmentally controlled conditions (temperature, incident light, [CO_2_], and air humidity) with our recently developed exposure chambers allowing for distinguishing the above-ground organs from soil and root system. In addition, we analyzed the VOC contents in leaves and roots and tested the effect of light on foliar emissions to better understand the origins of the emitted VOCs.

## 2. Results

### 2.1. Aboveground VOC Production and CO_2_/H_2_O Gas Exchange

**Figure 1:**
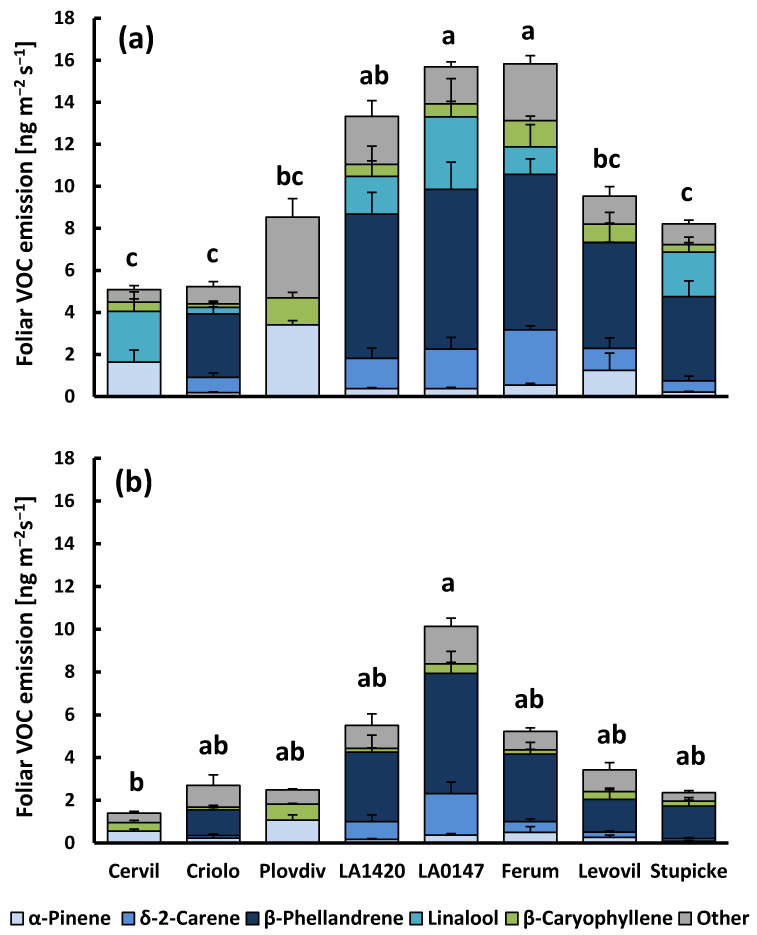
Foliar Volatile Organic Compounds (VOC) emissions of the eight accessions of the MAGIC tomato population under light (**a**) and dark conditions (**b**). Cervil, Criolo, Plovdiv24A (referred to as Plovdiv) and LA1420 belong to the *Solanum lycopersicum cerasiforme* group with cherry-type fruits, while LA0147, Ferum, Levovil and Stupicke Polni Rane (referred to as Stupicke) belong to the *Solanum lycopersicum lycopersicum* group with large fruits. Column sections show the mean emissions + SE (*n* = 4–6) of the five major emitted compounds and the sum of all other compounds. Superscript letters denote significant differences (*p* < 0.05) between genotypes for the sum of VOC emissions based on Bonferroni tests following an ANOVA (*p* < 0.001) and Dunn’s test following a Kruskal–Wallis test (*p* = 0.022) for light and dark VOC emissions, respectively (see supplementary material Table S2 for more details).

Tomato foliage emitted VOCs in low quantities, despite its aromatic character. The most abundant, regularly emitted ones were the monoterpenes β-phellandrene, δ-2-carene, linalool, α-pinene, and the sesquiterpene (*E*)-β-caryophyllene (Figure 1, supplementary material Tables S1 and S2). The five major compounds emitted under light conditions represented between 55 and 85% of the total emission, depending on the genotype. The remaining was composed of 10 monoterpenes (or derivatives), including β-pinene, myrcene, α-phellandrene, α-terpinene, *p*-cymene, β-ocimene, γ-terpinene, terpinolene, (*Z*)-2,6-dimethyl-2,6-octadiene), and four sesquiterpenes, namely δ-elemene, α-humulene, and germacrene-d, plus one unknown sesquiterpene. These minor emissions were more or less close to the detection limit of our system and, therefore, not consistently found in all of the replicate measurements.

At 30 °C and lighted conditions, the mean rate of total VOC release was 10.2 ± 1.5 ng m^−2^ leaf area s^−1^ (1.87 ± 0.31 µg g^−1^ leaf dry weight h^−1^) across all of the genotypes. The emission rates (sum of total VOCs and sum of main VOCs) were significantly different between genotypes (*p* < 0.001, Figure 1a and Supplementary material Table S2), while there was no genotypic effect on CO_2_-H_2_O gas exchange parameters apart from transpiration (*p* = 0.023, Cervil > Criolo, data not shown). Genotypic differences in VOC emissions were mostly due to variability in the emissions of monoterpenes (the sum of monoterpenes, *p* < 0.001), and less to variability in sesquiterpene emissions (sum of sesquiterpenes *p* = 0.087). Regarding the main compounds, three cherry tomato genotypes from the *cerasiforme* group (Cervil, Criolo and Plovdiv) had consistently lower emissions than all other genotypes, with β-phellandrene and δ-2-carene being absent in the emissions of Cervil and Plovdiv. However, Cervil and Plovdiv emitted α- and β-pinene at much higher rates than the other genotypes that are dominated by high emissions of β-phellandrene and δ-2-carene (mean α-pinene emissions of 2.43 ± 0.46 and 0.45 ± 0.14 ng m^−2^ s^−1^ for Cervil and Plovdiv, and other genotypes, respectively). Linalool emissions were highly variable and they occurred rather sporadically in all genotypes. When released, linalool emissions scaled positively with the foliar CO_2_-assimilation rate (R^2^ = 0.44, data not shown). The linalool emissions did not correlate to the emission of any other VOC. On the contrary, the emissions of all other compounds correlated to each other in a given VOC class (see Supplementary material Figure S1 for examples).

When plants were darkened (Figure 1b), the total aboveground VOC emission was reduced by 60%, on average, when compared to light conditions (4.2 ± 1.0 ng m^−2^ s^−1^ and 0.71 ± 0.17 µg g^−1^ h^−1^). Darkening reduced emissions of all genotypes, albeit the effect was not significant for LA0147, whose dark emissions largely scattered among replicates. Sesquiterpene emissions were generally less reduced by darkness than monoterpenes (40% vs. 65%) and not significantly affected in seven of eight genotypes. Looking at individual VOCs, most of the main and trace compounds were still released in darkness, except linalool, β-pinene, and (*Z*)-2,6-dimethyl-2,6-octadiene, which could never be detected in the absence of light, regardless of the genotype (Figure 1b, Supplementary material Table S2). Dark emissions were different among genotypes, although the differences were less significant than for light emissions (*p* = 0.022 and 0.021 for total VOC and sum of major VOC emissions). Pairwise comparisons of genotypes revealed that only the dark emissions of Cervil and LA0147 were significantly different one from each other. Nevertheless, the pattern of genotypic variability of dark emissions was similar to that of light emissions.

Foliar VOC contents that were gained from solvent extracts showed that most, although not all, emitted VOCs are stored in tomato leaves (Table 1). Among the main emitted VOCs, only linalool was absent in the leaf extracts. By contrast, β-phellandrene, δ-2-carene, α-pinene, and (*E*)-β-caryophyllene were consistently found in leaf extracts, even in the genotypes that hardly emitted them. In addition, two apparently non-emitted phenolic compounds were present in the leaf extracts (methyl-salicylate and eugenol) though not always. The mean VOC contents across the eight genotypes were 69.3 ± 9.8 µg g^−1^ leaf fresh weight (range: 43.5 ± 5.6–95.6 ± 18.1 µg g^−1^) for the sum of all VOCs and 56.8 ± 8.6 µg g^−1^ (range 30.6 ± 4.5–90.3 ± 16.7 µg g^−1^) for the sum of main compounds (Table 1). VOC contents that were extracted from individual leaf samples were quite variable. Nevertheless, the plant’s genotype had a significant effect on the VOC contents of tomato leaves (*p* = 0.04). As for emissions, this genotypic variability was mainly due to differences in monoterpene contents (ANOVA, *p* = 0.002 for the sum of monoterpenes). Especially, β-phellandrene contents were markedly different among genotypes, with lowest contents being observed for Cervil, Criolo, and Plovdiv in accordance with their emissions. The leaf concentrations of individual VOCs were hardly correlated to each other. The best correlations were observed between β-phellandrene and δ-2-carene contents (R^2^ = 0.44), and between methyl-salicylate and δ-elemene contents (R^2^ = 0.33; data not shown).

### 2.2. Belowground VOC Production

**Figure 2:**
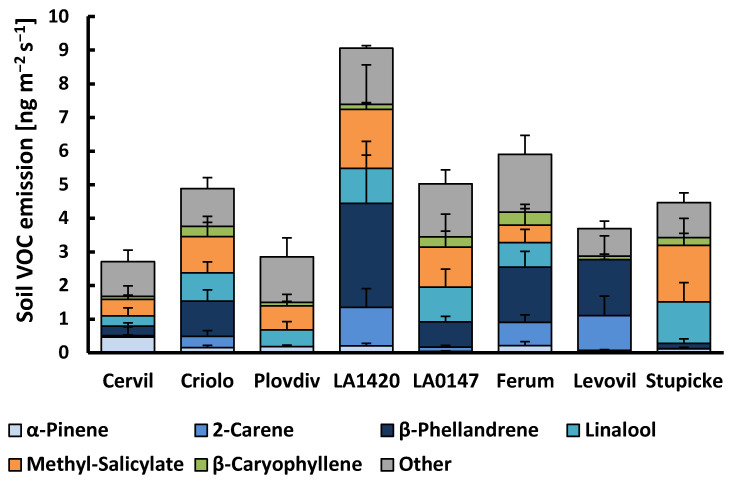
VOC emissions from the soil surface of the eight accessions of the MAGIC tomato population. Column sections show the mean emissions + SE (*n* = 3–4) of the six major emitted compounds and the sum of all other compounds. There was no significant difference between genotypes for individual VOCs or the sum of VOC emissions (Kruskall–Wallis tests, *p* > 0.05).

About 20 VOCs released from the soil surface were associated with the presence of plants, among which all of the monoterpenes and sesquiterpenes that were observed in the aboveground emissions (Figure 2, Supplementary material Table S1). In addition, methyl-salicylate and an unidentified monoterpene occurred in the soil emission, as well as various oxygenated VOCs, including dodecanal, tetradecanal, methyl-heptanone, hexanoic acid, and 3-octenol. However, the latter group of oxygenated VOCs, as well as linalool and the sesquiterpene δ-elemene, were also frequently detected in the emissions of soil blanks (without plant) indicating that a variable fraction of these VOCs emitted from the soil surface was not exclusively linked to the presence of plants. Belowground emissions, either expressed per soil surface or per root dry weight, scattered strongly among plant replicates in all genotypes. Consequently, no significant genotypic effects were observed neither for the sum of VOC emissions nor for individual VOC classes. Overall, the mean total VOC belowground emissions amounted to 4.86 ± 0.64 (± SE, *n* = 34) ng m^−2^ ground surface s^−1^ or 0.53 ± 0.07 µg g^−1^ root dry weight h^−1^. There were distinct co-variations in the appearance of individual VOCs. β-Phellandrene, δ-2-carene, α-terpinene, α-phellandrene, *p*-cymene, and terpinolene emissions correlated well with each other (R^2^ > 0.7). By contrast, these VOCs were only moderately correlated with the emissions of myrcene, γ-terpinene, and β-ocimene (R^2^: 0.3–0.6), and not at all with the emissions of α-pinene, linalool, sesquiterpenes, and other VOCs (Supplementary material Figure S2). Instead, the linalool soil emissions scaled positively with the emission of methyl-salicylate (R^2^ = 0.86) and with the emissions of sesquiterpenes, especially germacrene-D (R^2^ = 0.81), while α-pinene emissions were not related to other VOCs other than β-pinene. The variability in the VOC emission rates per soil surface were mostly unrelated to the root dry weights of the plants. Among individual VOCs, only the soil emissions of myrcene, β-ocimene, linalool, and methyl-salicylate scaled positively with root dry weights (respective R^2^ discarding zero emission values: 0.75, 0.49, 0.38, 0.29; data not shown).

Analyses of root extracts revealed the presence of six VOCs in low concentrations, of which only β-phellandrene and methyl-salicylate were also found in the soil emissions. Two benzenoïds phenyl-acetaldehyde and guaiacol, the acyclic monoterpene alcohol geraniol and one unidentified aldehyde, were the other stored VOCs inside roots (Table 2). The two major stored compounds, phenyl-acetaldehyde and methyl-salicylate, scaled positively with each other (R^2^ = 0.77) and to a lesser extent with guaiacol (R^2^: 0.52 and 0.30, data not shown). There were no apparent correlations between the amounts of the other stored VOCs. As for belowground emissions, the VOC contents from root extracts were highly variable among plant replicates and, hence, no significant genotype effect could be seen from our data. On average, the total amount of VOCs stored in the roots was 2.55 ± 0.38 µg g^−1^ FW.

### 2.3. Covariations between Genotypic Differences in above and Belowground VOC Production

**Figure 3:**
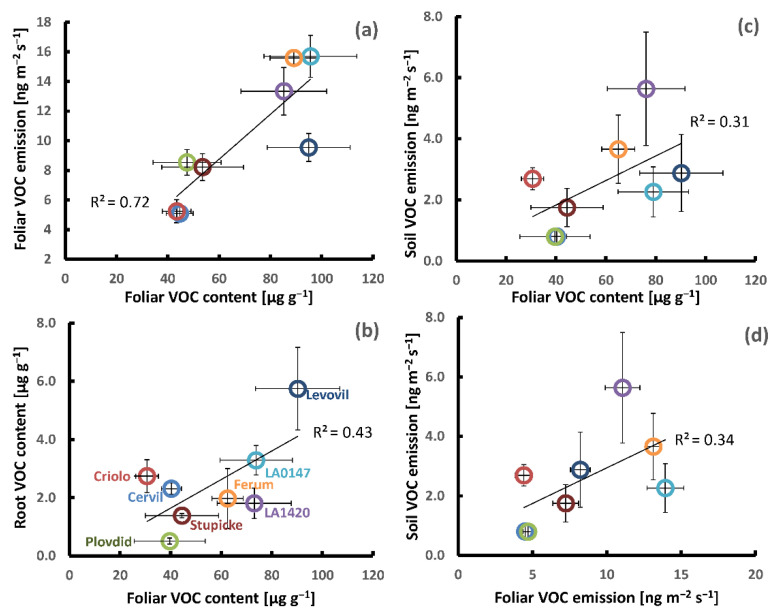
Examples of genotypic covariations in the aboveground and belowground VOC production of the eight parents of the MAGIC tomato population; (**a**–**c**): Foliar VOC contents vs. foliar VOC emissions, root VOC contents, and soil VOC emissions; and, (**d**) foliar VOC emissions vs. soil VOC emissions. The small inserted graph in (**a**) shows the foliar VOC contents vs. foliar CO_2_-assimilation. All of the data points represent the mean values per genotype ± SE of three to six plant replicates. Note that the belowground VOC production was determined on different plants than those used for the determination of aboveground VOC production.

Based on the mean values per genotype, we performed Pearson correlation analyses in order to see whether genotypic differences in the above and below ground production of VOCs were linked to each other (Table 3, Figure 3). A clear positive correlation was seen between the genotypic variations in foliar VOC emission and foliar VOC contents for the sum of all VOCs (Figure 3a) and the sum of major VOCs, especially if the genotype Levovil was left out. The correlations between contents and emissions of individual VOCs were more scattered than the sum of VOCs and β-phellandrene showed the best fit (R^2^ = 0.53, data not shown). Using the emission data set that was obtained under dark conditions did not improve the correlations to foliar VOC contents. The mean leaf VOC contents per genotype were positively correlated with the mean CO_2_-assimilation rates per genotype (R^2^ total VOCs: 0.52, R^2^ major VOCs: 0.70 (Figure 3a, small inserted graph)), whereas there was no clear relationship between the genotypic differences in foliage VOC emission and CO_2_-assimilation (R^2^ < 0.3, data not shown).

The mean soil emissions per genotype were unrelated to their root VOC contents either for the sum of VOCs (Table 3) or for individual common VOCs. On the other hand, the mean soil emissions per genotype scaled weakly with mean foliar VOC emissions and contents (Table 3, Figure 3c,d). This positive relationship was mainly associated with monoterpene emissions and less with other VOC classes. Among the individual commonly emitted VOCs, the mean myrcene, *p*-cymene, and β-phellandrene aboveground emissions showed the best correlations with the respective mean belowground emissions (R^2^ = 0.75, 0.44 and 0.43, respectively, data not shown). Further, no or only traces of β-phellandrene and δ-2-carene were released from the soil surface of the genotypes Cervil and Plovdiv consistent with their absence in the foliar emissions. The mean VOC root contents per genotype were also uncorrelated with mean foliage emissions, while they weakly correlated with mean VOC leaf contents (R^2^: 0.30 and 0.43, Table 3). However, this correlation should be considered with caution, since it was mainly based on Levovil, showing strong VOC contents in both leaves and roots (Figure 3b). Concerning the two individual compounds stored in both organs, only the mean foliar contents of β-phellandrene were weakly correlated with the mean β-phellandrene contents in the roots (R^2^: 0.30, data not shown), whereas the mean methyl-salicylate contents in roots were unrelated to those in leaves (R^2^: 0.0 and 0.26 after the removal of Levovil, data not shown).

## 3. Discussion

### 3.1. Aboveground Emissions

The present study revealed that constitutive VOC emissions of intact tomato foliage are mainly composed of monoterpenes and a few sesquiterpenes that are only released at low rates ranging between 5 and 20 ng m^−2^ s^−1^. VOC emissions from tomato foliage have already been described in various previous studies and the composition and quantity of constitutively released VOCs reported therein are consistent with those observed in our study [4,17,20,22,26].

The majority of VOCs were emitted under both light and dark conditions and they were also found in the leaf extracts, with leaf contents roughly scaling with emission rates. This strongly suggests that emissions predominantly stem from storage pools that are localized in the glandular trichomes that cover all aboveground organs. Especially, type VI tomato trichomes produce large amounts of volatile terpenes, which are stored inside an intercellular cavity that is located in the middle of the four secretory apical cells [31]. However, the emission rates of all stored terpenes were reduced under darkness, which is unexpected if emissions are exclusively governed by the physical diffusion from the apical cavity to the atmosphere. A possible explanation of this partial light dependency is that another independent terpene source in the green tissues of tomato foliage exists, whose pool size is small and emission is directly linked to a de-novo-biosynthesis requiring photosynthetic input of primary carbon-substrates and biochemical energy. Alternatively, the partial light dependency comes from the intensive terpene production in young trichomes occurring during the growth and maturation of leaves and stems. Glandular trichomes undergo distinct developmental stages, starting from gland initiation in the presecretory stage to the secretory stage, during which the storage space is filled, followed by the postsecretory stage [32]. During the secretory stage, the cavity lining that prevents the escape of stored metabolites gradually develops, so that a considerable fraction of terpenes transported to the storage space can escape into the atmosphere. Furthermore, a light dependency of this terpene production is plausible, because Tomato type VI trichomes cells contain functional, photosynthetically active chloroplasts, whose products, along with sucrose, imported from source tissues sustain the biosynthesis of secondary metabolites [33].

If different singular terpenes come from a same source, their emissions are more likely to be correlated than if they come from different independent sources. In our study, the foliar emissions of all stored monoterpenes were correlated to each other. In particular, β-phellandrene, δ-2-carene, and α-terpinene emissions were highly correlated, which is consistent with their common production by a single tomato terpene synthase (TPS20) that is predominantly active in the plastids of young and mature leaf and stem glandular trichomes [34]. Less good, although still significant, were their correlations with the emissions of α-pinene, which is produced in the same tissues, but by another plastidic enzyme (TPS9). By contrast, none of the emissions of the main monoterpenes stored were related to the sporadic emissions of linalool. In tomato, several plastidic and cytosolic enzymes that are not predominantly expressed in young glandular trichomes can synthesize linalool [33]. Several studies have reported that linalool production is induced by biotic stress elicitors [35,36,37,38]. Ref. [35] found that induced linalool emissions from tomato foliage followed a strict daily pattern, in agreement with the absence of linalool in dark emissions and in the leaf extracts that were observed in our study.

In summary, we conclude that, with the exception of linalool, all of the aboveground emissions of major monoterpenes were related to their constitutive production in glandular trichomes, whose light-dependent fractions presumably originated from trichomes in the intensive secretion stage that is principally present on young, immature leaf, and stem surfaces. This implies that, once trichomes enter the postsecretory stage, the stored VOCs are efficiently sealed inside the cavities and only diffuse out at tiny rates, because the fraction of young foliage present in our enclosures (ca. 20–25%) was small when compared to the degree of the emission’s light dependency (ca 50% for stored monoterpenes). The tight sealing helps to preserve the trichome’s integrity and, hence, ecological functions. The cavities easily break upon mechanical stress that is caused by herbivores and produce huge VOC bursts, together with sticky, non-volatile exudates that together provide a chemical (deterrent or toxic) and physical barrier against herbivore attacks [39]. The intraspecific variability we could demonstrate in the present study, most notably reduced VOC levels in the *cerasiforme* group, was almost exclusively associated with monoterpenes that are known to be constitutively formed and stored in type VI trichomes. Therefore, it is conceivable that the observed chemodiversity in foliar terpene emissions and contents is due to genotypic variation in trichome densities with possible impacts on their stress resistances [40]. The trichomes of type VI are morphologically different among tomato species, with a lower storage capacity in cultivated tomato species when compared to wild species, which are more resistant to environmental stresses [41]. However, to date, only very few studies have compared the ecological performances of the MAGIC parental genotypes although they present a wide range of phenotypes at the leaf and fruit levels [29]. Their phenotyping under contrasted conditions suggested a higher tolerance to water or salinity stress in the *cerasiforme* group [42,43,44,45]. Thus, our study brings complementary information on the genotypes related to the diversity of VOC production and emissions from vegetative organs.

To our knowledge, only one study has investigated the intraspecific chemical variability of foliar VOC emissions from tomato with a focus on salt stress effects [22]. Instead, many studies have concentrated on the varietal diversity of VOCs that are released from the tomato fruit given that fruit aroma is an important component of quality [46,47,48]. Generally, the fruit volatile bouquet of tomato is mainly composed of derivatives of amino acids, fatty acids and carotenoids, whereas monoterpenes and sesquiterpenes are almost absent. This clear difference in the VOC production of tomato plant organs underlines their different ecological functions as attractants and repellents, which is also reflected in distinct human olfactory sensations perceiving the scent of tomato fruit to be pleasant and the odor of tomato leaves as unpleasant. Interestingly, [49] compared the flavor contents of Cervil and Levovil fruits and reported distinct higher eugenol levels in Levovil than in Cervil, which was consistent with the different leaf contents observed in our study.

### 3.2. Belowground Emissions

To date, volatile emissions from tomato roots have rarely been investigated. Ref. [50] collected VOCs from dissected frozen tomato roots and detected few terpenes in addition to methyl-salicylate and pyrazine-derivatives. More recently, [51] employed a headspace technique that was combined with passive VOC sampling on adsorbent cartridges to investigate VOC release from intact tomato roots in soil. These authors detected a wealth of alkanes, alkenes, ketones, acids, phenolic, and unidentified compounds, plus several monoterpenes, but no sesquiterpene. However, both of the studies did not quantify the emission rates. In our study, the total amount of VOCs released from the soil surface varied between 1 and 10 ng s^−1^ m^−2^, with terpenes contributing more than half (monoterpenes, 52%; sesquiterpenes, 6%). An increasing body of literature is available on VOC fluxes from forest soils and, to a lesser extent, from agricultural soils (for an overview, see e.g., [52,53,54]). Monoterpene soil emissions were regularly recorded in ecosystems with terpene storing litter covering the ground. For example, in conifer forests, the reported emission rates vary between one-tenth and several hundreds of ng s^−1^ m^−2^ ground surface, depending on the forest and soil type, the amount and decomposition state of the litter, and the environmental conditions ([55], and references therein). The soil emission rates that we gained from potted plants fall in the same range, although no litter was present on the soil surfaces. Nevertheless, our observations indicate that the VOC emissions from the soil were partly associated with the aboveground VOC production of tomato plants: many VOCs emitted from the soil were found in the foliar VOC emissions and VOC leaf extracts, but not in the root extracts, and their emission rates varied independent of the root biomass of the plant replicate. In addition, the genotypic variation in soil VOC emissions was unrelated to the genotypic variation in root VOC contents, while it was weakly correlated with foliar VOC emissions and contents. On the other hand, the mean root VOC contents scaled positively with their foliar contents pointing to an inherent link between the plant’s capacity to produce VOCs in belowground and aboveground organs. Indeed, β-phellandrene and methyl-salicylate emitted from the soil were found in both root and leaf extracts. Furthermore, the individual soil emission rates of methyl salicylate as well as that of all acyclic monoterpenes (myrcene, ocimene, linalool), were correlated with the root biomass of the plants, implying a root origin, especially when considering that the correlations between emissions from the root and from the soil surface have been likely weakened by unknown VOC retentions and transformations that occurred during path through the soil [10].

Thus, our findings let us conclude that VOC emitted from the soil surface consisted of a mix of volatiles that are derived from both above and belowground sources. Links between soil emissions and aboveground VOC production can be explained by VOC contamination of the upper soil layer by formerly decaying leaves (cotyledons) or disruption of glandular trichomes during plant handling and the subsequent adsorption of gaseous VOCs on soil particles or dissolution on aqueous surfaces. Furthermore, VOCs or their precursors might be transported between the above and belowground organs via the sap flow [56,57]. In addition to these aboveground VOC sources, soil emission also included VOCs originating from root production having no or only small pool sizes when compared to the aboveground organs. There is increasing evidence that roots and their associated microbiota exchange volatile metabolites that shape interactions with other soil organisms [10] and even affect root development, possibly by altering stress signaling [58]. The VOCs that are produced by roots can be similar to those produced by other organs or they can be organ specific. For example, isoprene, a major plant VOC typically formed in leaves of poplar, is also produced by roots albeit at much lower rates than in leaves [59]. In tomato, [34] analyzed the terpene profiles of different tissues and reported relatively high amounts of geraniol and β-phellandrene in roots, which coincides with the terpenes are that present in our root extracts. A large number of terpene synthases are expressed in tomato roots, although their putative products are not always detectable in root tissues [34,60]. According to our soil emission data, one or several of them produce acyclic monoterpenes without relevant accumulation in root tissues.

### 3.3. Implications for Crop Surveillance

Most of the tomato production that is intended for the consumption of fresh fruits is grown in greenhouses. Innovative methods for early disease detection in crop survey are needed to foster environmentally and human-friendly agriculture. Online monitoring of VOC exchanges from crops might be a promising tool, especially in greenhouse culture, where environmental drivers affecting the atmospheric VOC load are considerably reduced and more controlled than in outdoor conditions. Several pilot studies have already endeavored to develop this application in tomato culture [16,18,19], and our results confirm the suitability of this crop as a model species: constitutive emissions from tomato foliage are very low under undisturbed conditions, i.e., one to three orders of magnitude lower than those of other typical monoterpene emitting vegetation, such as conifers and Mediterranean oak species [61]. The two main compounds β-phellandrene and δ-2-carene do not figure among the monoterpenes that are commonly observed in the air [62]. Therefore and because the trichome cavities wherein these compounds are stored can easily break, any increase in the greenhouse air concentrations outside cultural practices can indicate the presence of pests, in particular chewing arthropods and necrotrophic pathogens [20,63].

However, our results revealed several issues that can interfere with the interpretation of temporal variations in constitutive VOC pattern. First, we observed clear quantitative and qualitative differences between the foliage emissions of the eight genotypes. Cherry tomato accessions tended to have lower emission capacities and two of them mainly released pinenes, which are commonly found in the atmosphere. Thus, the suitability and usefulness of VOC monitoring in tomato culture can depend on the cultivar. Second, besides temperature, light was identified as an environmental factor driving temporal changes in VOC emissions. We hypothesized that the emissions from young immature foliage are mainly light dependent on the contrary to the emissions from mature foliage. If so, the degree of light dependency to be considered in greenhouse VOC monitoring will change during the crop cycle depending on the proportions of young and mature tissues. Finally, we found that the soil substrate, together with roots, can represent a non-negligible VOC source, since the emission rates per soil surface were almost as high as the leaf emission rates, but more variable. In addition to leaf constitutive VOCs, relatively high levels of methyl-salicylate and linalool were detected in soil emissions, both being typical stress-induced VOCs [64]. Linalool was also emitted from foliage in highly irregular amounts, possibly pointing to the presence of stress, although there was no visible sign of pests. However, our plants were grown in a non-sterile environment, so that a low presence of microorganisms eliciting some stress reactions cannot be ruled out. Further studies on belowground VOC emissions are needed in order to understand their origins and links with aboveground VOC sources to assess whether the quantities that are released under real cultural condition may blur or oppositely complement the diagnostic value of VOC markers from foliage emissions.

To conclude, our study provides new insights into the genotypic variation of constitutive VOC production in cultivated tomatoes, complementary to several previous studies that have phenotyped the MAGIC parents at the fruit level. The main purpose of this study was to correctly quantify the emissions under non-stress conditions and assess their origins and variations. It is a first essential step toward the use of VOCs for developing agro-ecological applications in greenhouse production.

## 4. Materials and Methods

### 4.1. Plant Material

**Figure 4:**
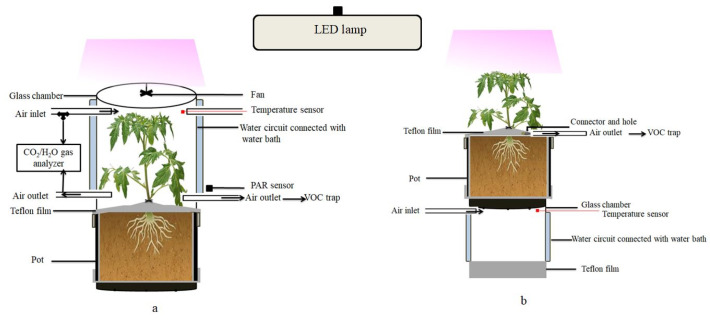
Schematic of the dynamic enclosure system used for the measurement of VOC emissions from the entire foliage (**a**) or from the soil surface (**b**) of tomato plants. The arrows indicate the direction of the airflow. The double walled middle section of the chamber with the temperature-regulated water circuit is shown in blue.

INRAE Centre for Vegetable Germplasm (CRB-Leg, Avignon, France) provided the seeds of the eight parents of the MAGIC tomato population and it grown in a glasshouse at day/night temperatures of 22/18 °C. Several batches of plants were grown regularly over the experimental period in order to have plants at a similar developmental stage all of the time. After about three weeks, individual seedlings were transferred to home-made cylindrical PVC pots of ca. 3 L volume filled with a mixture of sand and potting soil (Substrat SP178, Klasmann-Deilmann, France) and then regularly irrigated with a nutrient solution (Liquoplant Rose, 5 mL L^−1^ deionized water). The inner walls of the pots were lined with FEP films (50 µm thick), which were folded twice over the edge on the upper open side of the pot and fixed with a silicone sealing ring, leaving approximately 10 cm of FEP film protruding (Figure 4). Before the experiment, the protruding film was equipped with a two-way PTFE stopcock (BOLA, Bohlender GmbH, Grünsfeld, Germany) and closed around the stem base to separate the upper foliage air space from the pot with soil and roots.

### 4.2. Enclosure System and VOC Emission Measurement Protocol

All of the measurements were made on vegetative plants of 16 to 18 cm height (about seven to eight weeks after sewing, five to six fully expanded leaves and no flowers). The VOC emissions were measured with a dynamic headspace system operating two identical glass chambers fixed on a lifting platform side-by-side (for further description see [65]). Each chamber consisted of an open double walled glass cylinder of 16 cm diameter (same diameter as the pots) and 21 cm height (volume 4.2 L) featuring six inlet/outlet ports to the inner air space plus two ports to circulate thermostatic water from a water-bath inside the double glass wall (Figure 4). The chambers were closed on the top by removable glass lids holding a Teflon fan (motor outside) to ensure the homogenous mixing of the air inside the chambers. A programmable temperature controller regulated constantly the temperature of the water bath to a target air temperature of 30 °C measured by an inserted thermocouple (Chrom-Constantan, OMEGA, Biel, Switzerland). Additional thermocouples were mounted in order to survey the chamber air temperatures. Photosynthetic Photon Flux Density (PPFD) was measured by two quantum sensors (LI-COR, PAR-SB 190, Lincoln, NE, USA) fixed outside the chambers. A LED lamp (LX60 Heliospectra AB, Göteburg, Sweden) illuminated the chambers with a PPFD of 600 ± 30 µmol m^−2^ s^−1^. Temperature and PPFD data were recorded by a 21× Campbell data logger (Campbell Scientific Ltd., Shepsherd, UK).

For measuring the aboveground emissions (Figure 4a), the plants were mounted inside the open chambers (lids removed) in the evening the day before VOC measurements. The lower border of the glass chamber was precisely placed on the upper border of the pot and sealed outside with a silicone O-ring. The plants remained in the open chambers overnight at room temperature in order to ensure that any VOC emission bursts that were caused by the handling were gone [25]. The plants were illuminated with a program simulating sunset and sunrise with respect to outdoor conditions. The next day, chambers were flushed with artificial dry air (Alphagaz, 2 Air, Paris, France) at a constant rate of 1 L min^−1^ regulated by mass flow controllers (Mass Stream, M + W Instruments GmbH, Leonhardsbuch, Germany). Next, the chambers were closed, the air temperature was set to 30 °C and pure CO_2_ (Air liquid, CO_2_ N45) was injected in the inlet air stream via high precision mass flow controllers (El-Flow Select, Bronkhorst France SAS, Montigny-les-Cormeilles, France) to achieve CO_2_ concentrations of 400 ± 20 ppm inside the chambers. If necessary, the air was humidified by passing an adjustable fraction of the dry artificial air via a bypass through a gas washing bottle filled with distilled water in order to maintain a relative air humidity between 60% and 70%. The CO_2_ and H_2_O concentrations of the air entering and leaving the chambers were continuously monitored with infrared gas analyzers (LI-COR 7000 combined with LI-COR 840, Lincoln, NE, USA). The VOC emissions were measured after an acclimatization period a 90 min. VOCs were sampled on stainless steel cartridges (Perkin Elmer, Villebon, France) containing porous adsorbing polymers of Tenax TA and Carbotrap B in about equal quantities. The cartridges were connected to the chamber inlet and outlet ports and air was drawn through the adsorbent for 40 min at a constant flow rate of 150 mL min^−1^ by means of a pump and a mass flow controller. After this first measurement under light conditions, the lamp was turned off and the chambers were covered with a black sheet to determine VOC emissions under dark conditions. The temperature and CO_2_ concentrations were maintained at the same levels and VOCs were sampled after acclimation, as previously described. Subsequently, the plants were removed from the chambers and the foliage was collected in order to determine the VOC contents, total leaf dry masses, and surfaces. The leaf surface was determined by scanning leaves (Epson perfection V800) and processing the scans with Image J5 software (National Institutes of Health, Bethesda, MD, USA). The foliar dry weights were measured with a microbalance (Sartorius CP224S) after oven-drying at 60 °C for 72 h.

For measuring the belowground emissions (Figure 4b), the following protocol was applied: the pots with plants (different replicates than those used for foliage emission measurements) were mounted on the top of the open chambers (lids removed), while the open chamber bottom was closed with a sheet of FEP film hold by a silicon O-ring in order to ensure that only pure artificial air diffuses though the soil via the drain hole at the bottom of the pots. Air temperature and CO_2_ concentrations were adjusted to 30 °C and 400 ppm, respectively. After acclimation, an adsorbent cartridge was connected to the two-way PTFE stopcock inserted in the FEP film enclosing the headspace above the soil and VOCs were sampled at a flow rate of 150 mL min^−1^ for 60 min. The belowground emissions were measured once per plant replicate under illuminated conditions. Afterwards, roots were harvested to determine their VOC contents and dry weights.

In order to account for VOC background of the headspace system, VOC measurements were carried out using pots that were filled with substrate, but without plants, according to the same protocols as for plant aboveground and belowground emission determination.

### 4.3. Solvent Extraction of VOCs Stored in Leaves and Roots

The VOC contents were measured following the method that was described in [66]. Briefly, representative leaflets or root sections of each plant were carefully detached with a pair of scissors and plunged in 10 mL of dichloromethane after weighing the fresh sample on a microbalance (average fresh weights of leaves and roots were around 350 and 900 mg, respectively). The solution was inserted in an ultrasonic water bath for ten minutes, briefly vortexed, and then left for an hour at room temperature to complete extraction. Next, the plant material was removed from the extract and the extract was concentrated to a volume of approximately 250 μL in a flow of pure nitrogen after adding an internal standard of 40 µL biphenyl (0.1 mg mL^−1^).

### 4.4. GC-MS Analysis of VOC Emissions and Contents

VOCs that were sampled on adsorbent cartridges were analyzed by gas chromatography coupled with mass spectrometry (GC-MS) while using a Shimadzu QP-2010-SE instrument equipped with a TD-20 thermal desorption system (Shimadzu, Kyoto, Japan). Directly before analysis, cartridges were pre-purged for 5 min with pure nitrogen (flow rate ca. 30 mL min^−1^) to remove excessive water and were then thermally desorbed by flushing the heated tubes at 250 °C for 10 min with carrier gas (helium) at a rate of 30 mL min^−1^. The desorbed VOCs were trapped at −10 °C on a low-dead-volume cold trap containing a small amount of Tenax-TA. After desorption, condensed VOCs were thermally injected into the column by flush heating the cold trap to 250 °C for 5 min. The VOCs were separated on an Optima 5 MS column (length: 30 m, ID: 0.25 mm, FT: 0.25 µm; Macherey-Nagel, Düren, Germany) while using the following oven program: 2 min at 40 °C, 5 °C min^−1^ to 200 °C, 10 °C min^−1^ to 250 °C, kept for 4 min.

Analyses of solvent extracts were carried out with a Shimadzu GC-MS QP-2010-Plus that was equipped with the same column. An aliquot of 1 μL of the concentrated extracts was injected with a split ratio of 1:4 and the oven was run with the following temperature program: 40 °C for 1 min, 3.2 °C min^−1^ to 100 °C, 2.90 °C min^−1^ to 170 °C, 10 °C min^−1^ to 250 °C, held for 6 min.

GC-MS both used helium as carrier gas at a constant flow of 1 mL min^−1^. Transfer lines were kept at 200 °C and ion sources at 250 °C. MS acquisition was operated in scan mode (*m*/*z* range: 35–350), applying an ionization energy of 70eV. The peaks were identified by comparing their retention indices and mass spectra with those of commercial databases (NIST 2005, Wiley 2009, Adams, 2005, supplementary data Table S1) as well as pure standards dissolved in methanol (Sigma Aldrich^®^, Darmstadt, Germany). Standard solutions of various VOCs at three dilution levels were used in order to quantify emissions, namely α-pinene, *p*-cymene, linalool, methyl-salicylate, acetophenone, geranyl acetone, α-humulene, β-caryophyllene, octanal, and decanal. The calibration factor (peak area ng^−1^) of each VOCs was deduced from the slope of multipoint calibrations, revealing a good linear dependency of peak area to the respective compound amount. The mean calibration factor per compound class (i.e., monoterpenoïds, sesquiterpenoïds, phenolic compounds, and aldehydes) were applied to quantify the peaks identified in one of these classes, and the mean calibration factor of all the standards was applied to peaks to unidentified peaks.

The quantification of leaf and root VOC contents (solvent extracts) was exclusively based on the amount of the internal standard biphenyl, in order to take the losses of VOCs occurring during the concentration step into account.

### 4.5. Calculations and Statistical Analyses

Net CO_2_-assimilation, transpiration, and stomatal conductance of the enclosed foliage were calculated according to [67]. The foliage VOC emissions were calculated as the difference between the VOC concentrations in the chamber with plant and the concentration in the empty chamber, multiplied by the chamber air flow (1 L min^−1^) and divided by either the projected area or the dry weight of the enclosed foliage. In analogy, emissions from belowground organs were calculated as the difference between the VOC concentrations in the above soil headspace with plant and the concentration in the above soil headspace without plant, multiplied by the air exchange rate, which was assumed to be equal to the VOC sampling flow rate (i.e., 0.15 L min^−1^). The belowground emissions were expressed either per ground surface (pot cross-sectional area) or per root dry mass. Hence, the belowground emissions that are reported in our study do not represent true root emissions, but characterize the net VOC exchange at the soil-air interface associated with the presence of the plants.

Statistical analyses were carried out while using XLSTAT statistical software (v.7.0, Addin soft, Paris, France). The normality and homogeneity of data were assessed by the ShapiroWilk and Levene test. If the data showed normality and homogeneity, then the genotype effect was tested with a one-way ANOVA, followed by a Bonferroni post-hoc test for multiple pairwise comparison; otherwise, the non-parametric Kruskal–Wallis test combined with a Dunn test was applied. Paired Student tests or Wilcoxon signed-rank tests were used for the comparison of VOC emissions under light and dark. The differences between groups of measured variables were considered to be significant at a probability level of *p* < 0.05. Pearson correlation analyses were performed in order to test the covariation among variables. Consistency of correlations was visually checked by scatter plots. Correlations with a coefficient of determination R^2^ > 0.6 (more than 60% of covariation explained) are denoted as strong and those with 0.3 < R^2^ < 0.6 as weak. Correlations with R^2^ < 0.3 are considered to be meaningless.

## Figures and Tables

**Table 1:** VOC contents in leaf extracts (µg g^−1^ leaf fresh weight) of the eight tomato genotypes. Data are means of 4–6 plant replicates ± SE. The sum of the four major compounds represent 70% to 95% of the sum of all stored VOCs. Other VOCs include myrcene, γ-terpinene, δ-elemene, methyl-salycilate, and eugenol. ANOVA and Kruskal Wallis statistics were used to test whether means are different among genotypes. *p*-values indicate the overall probability of the test results (significant if *p* < 0.05). Superscript letters indicate which of the genotypes differs at α < 0.05.

VOC	Cervil	Criolo	Plovdiv	LA1420	LA0147	Ferum	Levovil	Stupicke	*p*
α-Pinene	3.9 ± 1.9	10.1 ± 1.7	1.0 ± 0.4	8.4 ± 3.2	9.0 ± 4.9	3.9 ± 1.2	6.6 ± 2.7	1.4 ± 0.6	0.101 ^$^
δ-2-Carene	10.4 ± 1.8	1.0 ± 0.6	2.3±2.4	6.7 ± 3.5	7.6 ± 3.8	1.4 ± 1.1	10.6±4.6	7.3 ± 5.9	0.323 ^$^
β-Phellandrene	12.9 ± 2.7 ^a^	13.5 ± 4.3 ^ab^	13.9 ± 4.3 ^ab^	52.2 ± 13.2 ^ab^	30.0 ± 10.3 ^ab^	42.9 ± 6.5 ^ab^	57.6 ± 11.8 ^b^	22.3 ± 4.8 ^ab^	0.002 ^£^
β-Caryophyllene	13.1 ± 2.3	5.9 ± 1.0	22.4 ± 9.5	5.8 ± 2.1	27.1 ± 7.8	14.3 ± 6.4	15.5 ± 4.4	13.4 ± 3.5	0.240 ^$^
Sum major VOCs	40.3 ± 4.0 ^a^	30.6 ± 4.5 ^a^	39.6 ± 14.0 ^a^	73.1 ± 14.7 ^a^	73.8 ± 14.3 ^a^	62.5 ± 6.2 ^a^	90.3 ± 16.7 ^a^	44.4 ± 14.5 ^a^	0.045 ^£^
Sum all VOCs	44.7 ± 5.3 ^a^	43.5 ± 5.6 ^a^	47.5 ± 13.2 ^a^	85.3 ± 16.8 ^a^	95.6 ± 18.1 ^a^	89.1 ± 9.3 ^a^	95.0 ± 16.2 ^a^	53.6 ± 15.9^a^	0.037 ^£^

^£^ ANOVA and Bonferroni post-hoc tests; ^$^ Kruskall–Wallis and Dunn’s post-doc tests.

**Table 2:** VOC contents in root extracts (µg g^−1^ root fresh weight) of the eight tomato genotypes. Data are means of 3–4 plant replicates ± SE. There was no significant difference between genotypes (*p* > 0.05; ANOVA on the sum of VOCs and Kuskall–Wallis tests on individual VOCs).

VOC	Cervil	Criolo	Plovdiv	LA1420	LA0147	Ferum	Levovil	Stupicke
β-Phellandrene	0.01 ± 0.01	0.02 ± 0.01	0.10 ± 0.02	0.08 ± 0.07	0.04 ± 0.02	0.01 ± 0.00	0.17 ± 0.02	0.00 ± 0.00
Geraniol	0.35 ± 0.13	0.09 ± 0.06	0.00 ± 0.00	0.16 ± 0.14	0.10 ± 0.09	0.08 ± 0.08	0.05 ± 0.02	0.03 ± 0.02
Methyl-salicylate	0.66 ± 0.09	1.04 ± 0.15	0.25 ± 0.14	0.50 ± 0.06	0.64 ± 0.29	0.66 ± 0.31	2.43 ± 0.59	0.50 ± 0.03
Phenyl-acetaldehyde	1.22 ± 0.16	1.47 ± 0.20	0.10 ± 0.01	0.99 ± 0.21	2.39 ± 0.96	1.18 ± 0.64	2.96 ± 0.88	0.75 ± 0.05
Unknown aldehyde	0.05 ± 0.02	0.05 ± 0.02	0.01 ± 0.01	0.17 ± 0.14	0.15 ± 0.05	0.06 ± 0.03	0.12 ± 0.05	0.08 ± 0.04
Guaiacol	0.07 ± 0.01	0.13 ± 0.02	0.06 ± 0.02	0.08 ± 0.04	0.12 ± 0.03	0.04 ± 0.02	0.14 ± 0.07	0.10 ± 0.01
Sum of VOCs	2.36 ± 0.30	2.79 ± 0.59	0.52 ± 0.15	1.97 ± 0.66	3.43 ± 0.50	2.03 ± 1.04	5.87 ± 1.44	1.46 ± 0.09

**Table 3:** Coefficients of determination (R^2^) of Pearson correlation analyses between foliar VOC emissions, foliar VOC contents, soil VOC emissions, and root VOC contents of the 8 tomato genotypes based on the mean values of three to six replicate measurements per genotype. Correlations with R^2^ ≥ 0.3 are considered as meaningful. If correlations between genotypes were obviously biased by a single outlier, R^2^ without outlier are given in parenthesis, together with the name of the genotype outlier (see also Figure 3).

Sum of All VOCs			
Foliar emission	0.73 (0.95 Levovil)	0.36	0.00
0.57 (0.89 Levovil)	Foliar content	0.25	0.30 (0.16 Levovil)
0.34 (0.61 LA0147)	0.31	Soil emission	0.00
0.04	0.43 (0.10 Levovil)	0.03 (0.23 LA1420)	Root content
Sum of major VOCs			

## Data Availability

Data are available from the authors on request.

## Supplementary Materials

The following are available online, Figure S1: Examples of correlations between individual VOCs emitted by tomato foliage, Figure S2: Examples of relations among soil emissions of individual VOCs, Table S1: List of VOCs analyzed by GC-MS that were observed either in the emissions from foliage and/or soil and/or in the extracts of leafs and/or roots, Table S2: Foliar emission rates of VOCs measured under light and dark conditions on the eight parents of the tomato MAGIC population.

## Sample Availability

Not available.
