# Supplementary material for: Insights into the Intraspecific Variability of the above and Belowground Emissions of Volatile Organic Compounds in Tomato
Source: Molecules. 2021 Jan 5;26(1):237. doi: 10.3390/molecules26010237 (PMC7796079; doi:10.3390/molecules26010237)
Supplement: Supplementary file 1 [file molecules-26-00237-s001.pdf]

Supplementary Material for

Insights into the Intraspecific Variability of the Above and Belowground Emissions of Volatile Organic Compounds In Tomato

Nafissa Dehimeche [1], Bruno Buatois [1], Nadia Bertin [2] and Michael Staudt [1,*]

[1] Centre d'Ecologie Fonctionnelle et Evolutive, CNRS-Université Montpellier-Université Paul-Valéry Montpellier–EPHE; Campus CNRS, F-34293 Montpellier cedex 5, France; nafissa.dehimeche@cefe.cnrs.fr (N.D.); bruno.buatois@cefe.cnrs.fr (B.B.)
[2] INRAE, UR115 Plantes et Systèmes de culture Horticoles, Site Agroparc, 84914 Avignon, France; nadia.bertin@inrae.fr
* Correspondence: michael.staudt@cefe.cnrs.fr; Tel.: +33 467613272

This file includes:
Supplementary Figures S1 and S2
Supplementary Tables S1 and S2

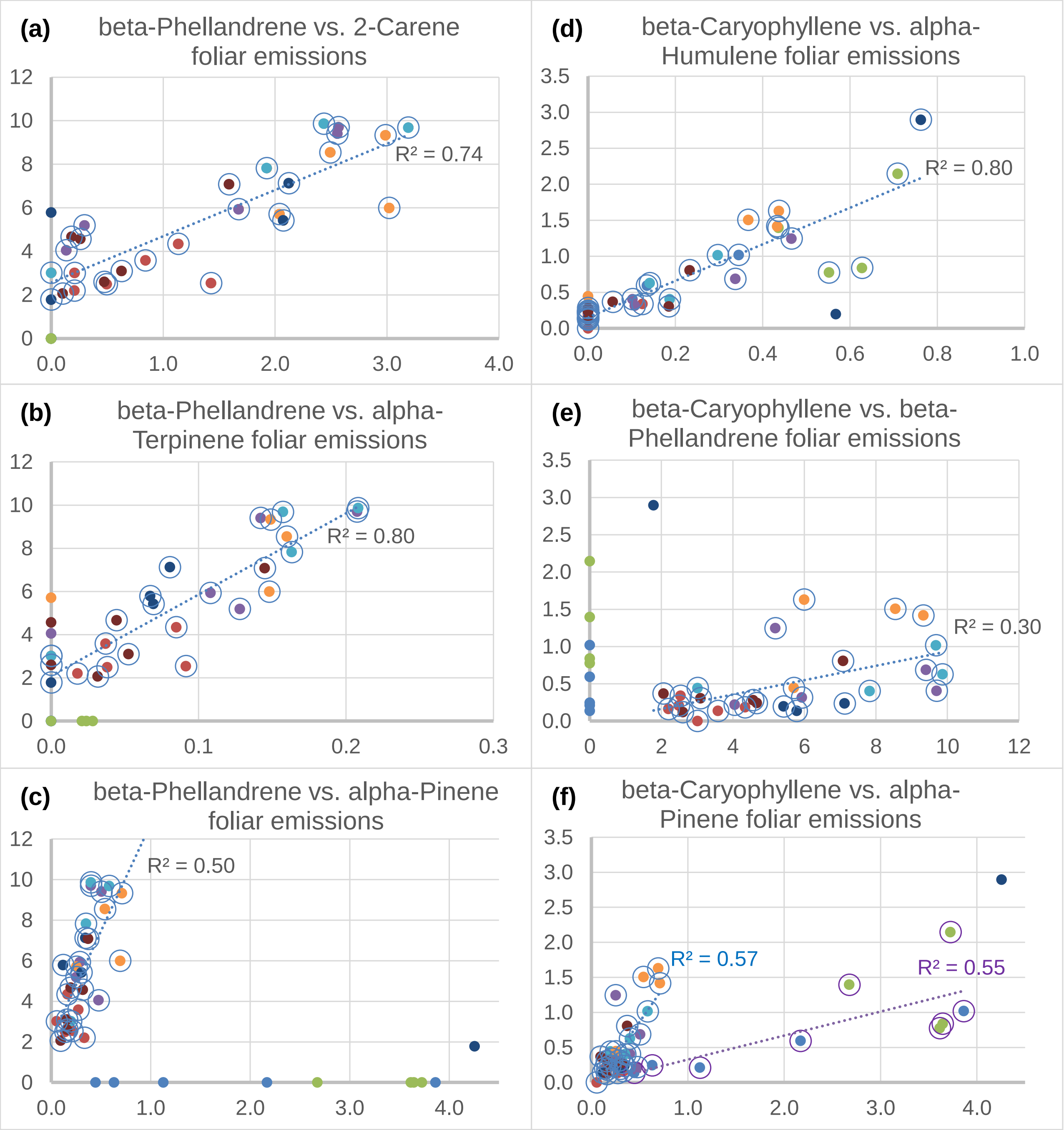

Figure S1. Examples of covariations between individual VOCs emitted by tomato foliage. Data points show measurements of single plant replicates across all genotypes (emission rates in ng m^{-2} projected leaf surface s^{-1}). Different colors correspond to the different tomato genotypes as shown in Figure 3(**b**). Lines and coefficients of determination R^2 show the results from Pearson correlations. Data points without open circles were excluded from the correlation analyses. Generally, the emissions of all individual monoterpenes were correlated with each other except linalool, whose emissions were unrelated to the emissions of other VOCs. The strongest correlations were found between the MTs β-phellandrene, δ-2-carene and α-terpinene (R^2: 0.70–0.80, examples shown in (**a**), (**b**)), whereas correlations with pinenes were more moderate (R^2: 0.50–0.57, example shown in (**c**)). The emissions of the major sesquiterpene β-caryophyllene were well correlated with the emissions of its structural isomer α-humulene (R^2: 0.80, (**d**)), and poorly or moderately with major monoterpenes ((R^2: 0.30–0.57, examples shown in (**e**), (**f**)). Note that two genotypes (Cervil, Plovdiv) did not emit β-phellandrene and δ-2-carene but larger amounts of α- and β-pinene compared to the other genotypes.

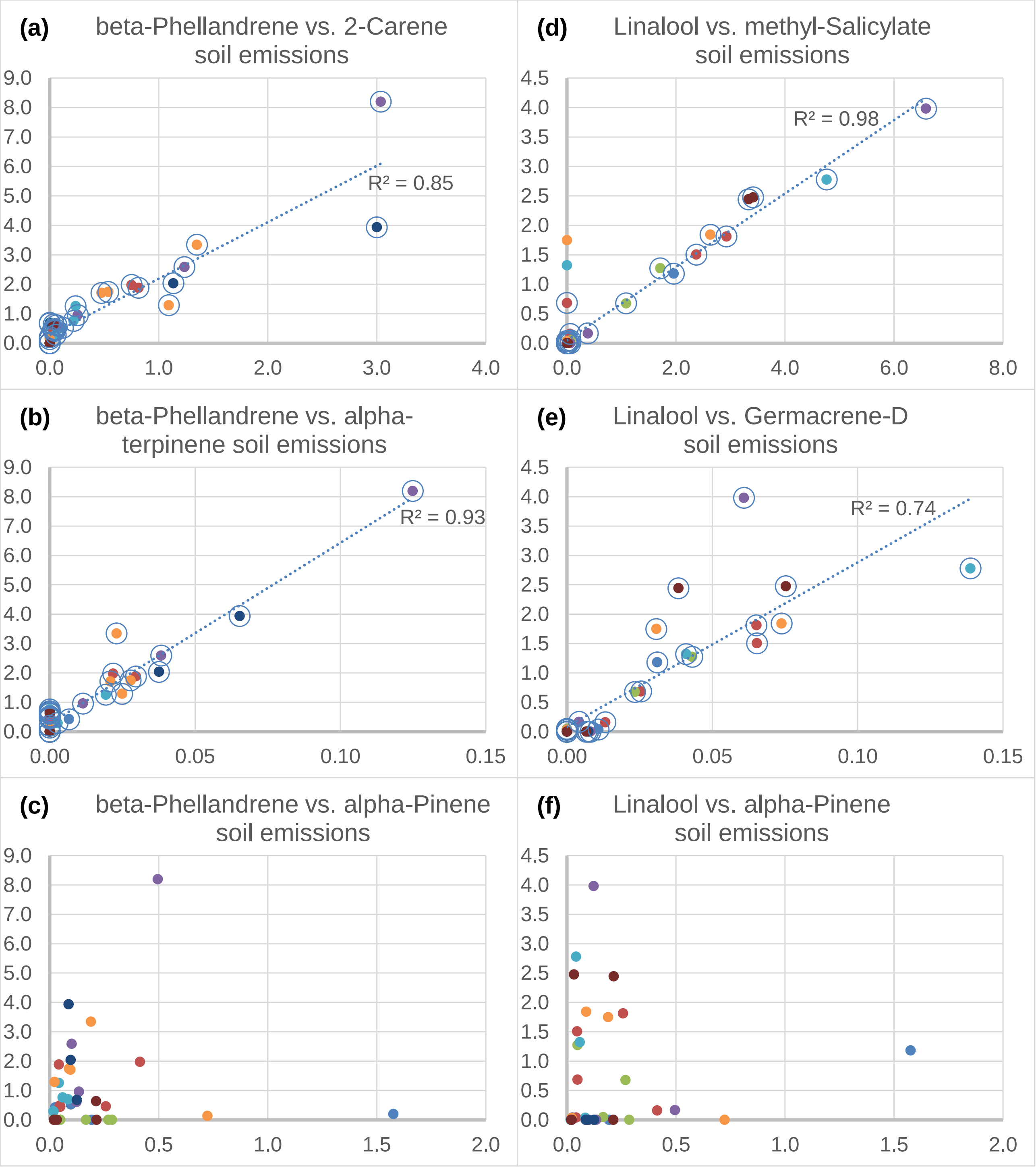

Figure S2. Examples of covariations among soil emissions (ng m^{-2} soil surface s^{-1}) of individual VOCs across all genotypes. Different colors correspond to the different tomato genotypes as shown in Figure 3(**b**). Pearson correlation analyses with resulting coefficients of determination R^2 were made on the data with open circles. Generally, there were two groups of VOCs, within each one individual emissions were correlated: group 1 consisted of β-phellandrene, δ-2-carene, α-terpinene, α-phellandrene, *p*-cymene and terpinolene, examples shown in (**a**), (**b**), and group 2 of linalool, methyl-salicylate and most sesquiterpenes examples shown in (**d**), (**e**). Soil emissions of α- and β-pinene were unrelated to other VOCs examples shown in (**c**), (**f**).

Table S1. List of VOCs analyzed by GC-MS that were observed either in the emissions from foliage and/or soil and/or in the extracts of leafs and/or roots. The occurrence of each compound is indicated below the compound name as follows: Emission from Foliage: E/F; Emission from Soil with Plant: E/S+P; Emission from Soil without Plants: E/S-P; Content in Leaves (leaf extract): C/L; Content in Roots (root extract): C/R. VOC classes: MT: non-oxygenated monoterpenes; oxMT: oxygenated monoterpenes; SQT: non-oxygenated sesquiterpenes; oxSQT: oxygenated sesquiterpenes; Phenolic: Phenolic compound (benzenoïds and phyl-propanoïds); OVOC: other oxygenated compounds. * Tentative identification; ND Not Detected; $Adams (2005), NIST 2005, Wiley 2009.

Compound (occurrence)	Formula (Molecular weight)	Assigned VOC class	Retention Time	Retention Index	Retention Time	Retention Index	Reference Retention Index$
			GC-MS analysis emissions		GC-MS analysis extracts		
Hexanal E/S-P	C6H12O (100)	OVOC	5.43	796	ND	ND	801
Nonene E/S-P	C9H18 (126)	OVOC	8.42	889	ND	ND	891
Heptanal E/S-P	C7H14O (114)	OVOC	8.7	898	ND	ND	901
α-Pinene E/F, E/S+P, C/L	C10H16 (136)	MT	9.81	933	11.02	939	932
3-Octen-ol* E/S+P, E/S-P	C8H16O (128)	OVOC	10.87	965	ND	ND	974
β-Pinene E/F, E/S+P	C10H16 (136)	MT	11.18	975	ND	ND	974
6-methyl-5-heptanone* E/S+P, E/S-P	C8H14O (126)	OVOC	11.44	983	ND	ND	981
β-Myrcene E/F, E/S+P, C/L	C10H16 (136)	MT	11.68	990	13.54	977	988
Octanal E/S-P	C8H16O (128)	OVOC	11.9	996	ND	ND	998
δ-2-Carene E/F, E/S+P, C/L	C10H16 (136)	MT	11.91	997	14.25	988	1001
α-Phellandrene E/F, E/S+P	C10H16 (136)	MT	12.08	1002	ND	ND	1002
unknown monoterpene E/S+P, E/S-P	C10H16 (136)	MT	12.3	1009	ND	ND	
α-Terpinene E/F, E/S+P	C10H16 (136)	MT	12.47	1015	ND	ND	1014
p-Cymene E/F, E/S+P	C10H14 (134)	MT	12.7	1022	ND	ND	1020
β-Phellandrene E/F, E/S+P, C/L, C/R	C10H16 (136)	MT	12.88	1027	15.5	1025	1025
Hexanoic acid E/S+P, E/S-P	C8H18O (130)	OVOC	12.89	1028	ND	ND	1027

Phenylacetaldehyde C/R	C7H8O (108)	Phenolic	ND	ND	15.68	1039	1036
β-Ocimene E/F, E/S+P	C10H16 (136)	MT	13.47	1046	ND	ND	1044
(Z)-2,6-dimethyl-2,6-Octadiene* E/F	C10H18 (138)	MT?	13.58	1050	ND	ND	978
γ-Terpinene E/F, E/S+P, E/S-P	C10H16 (136)	MT	13.82	1058	ND	ND	1054
Acetophenone E/S-P	C8H8O (120)	Phenolic	13.9	1060	ND	ND	1059
Octanol E/S-P	C8H18O (130)	OVOC	14.26	1069	ND	ND	1060
Terpinolene E/F, E/S+P	C10H16 (136)	MT	14.76	1087	ND	ND	1085
o-Guaiacol C/R	C7H8O2 (124)	Phenolic	ND	ND	17.59	1079	1087
Unknown Aldehyde C/R	C10H16O (152)	oxMT?	ND	ND	18.33	1094	
Linalool E/F, E/S+P, E/S-P	C10H18O (154)	oxMT	15.04	1096	ND	ND	1095
Nonanal E/S-P	C9H18O (142)	OVOC	15.11	1098	ND	ND	1100
unknown compound E/S+P, E/S-P	C10H20O (156)	?	15.53	1111	ND	ND	
Octanoic acid E/S-P	C18H16O2 (144)	OVOC	17.44	1175	ND	ND	1167
Methyl salicylate E/S+P, C/L, C/R	C8H8O3 (152)	Phenolic	ND	ND	22.99	1170	1190
Decanal E/S-P	C10H20O (156)	OVOC	18.14	1199	ND	ND	1201
Geraniol C/R	C10H18O (154)	oxMT	ND	ND	25.9	1228	1249
Nonanoic acid E/S-P	C9H18O (142)	OVOC	20.05	1262	ND	ND	1267
Undecanal E/S-P	C11H22O (170)	OVOC	21.06	1301	ND	ND	1305
δ-Elemene E/F, E/S+P, E/S-P, C/L	C15H24 (204)	SQT	22.11	1341	30.35	1335	1335
Eugenol C/L	C10H12O2 (164)	Phenolic	ND	ND	30.58	1350	1356
(E)-β-Caryophyllene E/F, E/S+P, C/L	C15H24 (204)	SQT	24.34	1426	34.93	1421	1417
Geranylacetone E/S-P	C13H22O (194)	oxMT	24.92	1449	ND	ND	1453

α-Humulene E/F, E/S+P, E/S-P	C15H24 (204)	SQT	25.2	1461	ND	ND	1452
Dodecanol E/S+P, E/S-P	C12H26O (186)	OVOC	25.51	1473	ND	ND	1469
Germacrene D E/F, E/S+P	C15H24 (204)	SQT	25.87	1487	ND	ND	1484
Pentadecane E/S-P	C15H32 (212)	OVOC	26.2	1500	ND	ND	1500
Dodecanoic acid E/S-P	C12H24O2 (200)	OVOC	27.66	1561	ND	ND	1565
Germacrene-4-ol* E/F	C15H26O (222)	oxSQT?	27.75	1565	ND	ND	1574
Unknown SQT E/F, E/S-P	C15H24 (204)	SQT	28.07	1578	ND	ND	
Tetradecanal E/S+P, E/S-P	C14H28O (212)	212	28.75	1606	ND	ND	1611

Table S2. Emission rates (mean ± SE, n = 4-6) of individual major VOCs, sum of major VOCs, sum of all monoterpenes, sum of all sesquiterpenes and sum of all compounds measured under light and dark conditions on the eight parents of the tomato MagicTom population. Please, refer to Table S1 above for the identification of minor monoterpenes and sesquiterpenes. P-values in the right column denote the significance levels for the effect of genotype based on ANOVA or Kruskall-Wallis tests (considered as signficant at $p < 0.05$). Superscript letters indicates the significant differences among the individual genotypes resulting from pairwise-comparisons with Bonferroni or Dunn tests. ND: Not Detected.

**VOC**	**Cervil**	**Criolo**	**Plovdiv**	**LA1420**	**LA0147**	**Ferum**	**Levovil**	**Stupicke**	**P-value**
α-Pinene	1.65 ±0.58^{bc}	0.20 ±0.04^{a}	3.42 ±0.20^{c}	0.38 ±0.05^{abc}	0.38 ±0.07^{abc}	0.55 ±0.09^{abc}	1.26 ±0.81^{abc}	0.22 ±0.04^{ab}	0.002[$]
	0.56 ±0.10^{a}	0.24 ±0.14^{a}	1.07 ±0.25^{a}	0.17 ±0.03^{a}	0.37 ±0.07^{a}	0.50 ±0.27^{a}	0.27 ±0.10^{a}	0.09 ±0.02^{a}	0.041[$]
δ-2-Carene	0.00 ±0.00^{a}	0.72 ±0.20^{ab}	0.00 ±0.00^{a}	1.45 ±0.48^{ab}	1.89 ±0.56^{ab}	2.63 ±0.19^{b}	1.05 ±0.50^{ab}	0.54 ±0.22^{ab}	0.002[$]
	0.00 ±0.00^{a}	0.11 ±0.07^{ab}	0.00 ±0.00^{a}	0.83 ±0.31^{ab}	1.94 ±0.54^{b}	0.50 ±0.14^{ab}	0.25 ±0.05^{ab}	0.12 ±0.05^{ab}	0.009[$]
β-Phellandrene	0.00 ±0.00^{a}	3.03 ±0.33^{ab}	0.00 ±0.00^{a}	6.86 ±1.04^{b}	7.60 ±1.30^{b}	7.39 ±0.74^{b}	5.03 ±0.93^{ab}	4.01 ±0.75^{ab}	<0.001[$]
	0.00 ±0.00^{a}	1.20 ±0.11^{ab}	0.00 ±0.00^{a}	3.26 ±0.79^{ab}	5.63 ±1.02^{b}	3.16 ±0.56^{ab}	1.53 ±0.45^{ab}	1.22 ±0.40^{ab}	0.002[$]
Linalool	2.42 ±0.92	0.30 ±0.30	0.00 ±0.00	1.78 ±1.44	3.45 ±1.82	1.31 ±1.07	0.02 ±0.01	2.11 ±0.72	0.246[$]
	ND	ND	ND	ND	ND	ND	ND	ND	-
(E)-β-	0.44 ±0.15^{ab}	0.17 ±0.05^{a}	1.29 ±0.26^{b}	0.57 ±0.17^{ab}	0.62 ±0.11^{ab}	1.25 ±0.22^{b}	0.87 ±0.55^{ab}	0.35 ±0.10^{ab}	0.007[$]
Caryophyllene	0.40 ±0.11^{ab}	0.14 ±0.04^{a}	0.75 ±0.04^{b}	0.17 ±0.02^{ab}	0.44 ±0.08^{ab}	0.21 ±0.02^{ab}	0.36 ±0.16^{ab}	0.23 ±0.06^{ab}	0.061[$]
Sum of major	4.51 ±0.45^{a}	4.42 ±0.59^{a}	4.70 ±0.33^{ab}	11.06 ±1.16^{ab}	13.93 ±1.24^{b}	13.13 ±0.54^{b}	8.21 ±0.67^{ab}	7.23 ±0.87^{ab}	<0.001[$]
VOCs	0.95 ±0.17^{a}	1.69 ±0.20^{ab}	1.82 ±0.23^{ab}	4.43 ±1.12^{ab}	8.38 ±1.64^{b}	4.37 ±0.82^{ab}	2.41 ±0.72^{ab}	1.96 ±0.45^{ab}	0.021[$]
Sum of Mono-	4.43 ±0.42^{c}	4.53 ±0.63^{c}	6.20 ±0.96^{c}	11.17 ±1.35^{ab}	14.69 ±1.29^{a}	13.48 ±0.38^{a}	7.95 ±0.68^{bc}	7.12 ±0.85^{bc}	<0.001[£]
terpenes	0.77 ±0.13^{a}	2.07 ±0.43^{ab}	1.13 ±0.26^{ab}	4.53 ±1.22^{ab}	9.39 ±1.81^{b}	4.85 ±0.94^{ab}	2.67 ±0.80^{ab}	1.79 ±0.48^{ab}	0.009[$]
Sum of Sesqui-	0.67 ±0.26^{a}	0.71 ±0.20^{a}	2.34 ±0.31^{a}	2.17 ±0.71^{a}	1.01 ±0.21a	2.10 ±0.36^{a}	1.60 ±0.80^{a}	1.10 ±0.21^{a}	0.087[£]
terpenes	0.63 ±0.15	0.63 ±0.24	1.36 ±0.04	0.98 ±0.46	0.74 ±0.13	0.38 ±0.03	0.74 ±0.26	0.57 ±0.14	0.422[$]
Sum of all	5.10 ±0.45^{c}	5.24 ±0.77^{c}	8.54 ±0.87^{bc}	13.34 ±1.60^{ab}	18.70 ±1.42a	15.59 ±0.44^{a}	9.55 ±0.95^{bc}	8.22 ±0.91^{c}	<0.001[£]
VOCs	1.40 ±0.26^{a}	2.70 ±0.66^{ab}	2.50 ±0.23^{ab}	5.51 ±1.56^{ab}	10.13 ±1.91b	5.23 ±0.98^{ab}	3.43 ±1.03^{ab}	2.36 ±0.47^{ab}	0.022[$]

[£] ANOVA plus Bonferroni post-hoc tests; [$] Kruskall-Wallis plus Dunn's test
